# Protein Homeostasis and Mitochondrial Quality Control in Neurodegeneration

**DOI:** 10.1002/wsbm.70014

**Published:** 2026-08-20

**Authors:** Rosa Di Lorenzo, Rola S. Zeidan, Riccardo Calvani, Vito Pesce, Emanuele Marzetti, Anna Picca

**Affiliations:** ^1^ Department of Biosciences, Biotechnologies and Environment Università Degli Studi di Bari Aldo Moro Bari Italy; ^2^ Department of Physiology and Aging, College of Medicine University of Florida Gainesville Florida USA; ^3^ Department of Health Outcomes and Biomedical Informatics, College of Medicine University of Florida Gainesville Florida USA; ^4^ Department of Geriatrics, Orthopedics and Rheumatology Università Cattolica del Sacro Cuore Rome Italy; ^5^ Fondazione Policlinico Universitario “Agostino Gemelli” IRCCS Rome Italy; ^6^ Department of Medicine and Surgery LUM University Casamassima Italy

## Abstract

Quality control (QC) processes include a network of cellular pathways that prevent the accumulation of toxic aggregates by repairing, recycling, and/or eliminating defective components, including mitochondria. Among these pathways are the proteostasis network, which regulates the proteome, and mitochondrial quality control (MQC) mechanisms, which maintain mitochondrial number and integrity. QC relies on a hierarchically and spatially integrated regulatory axis rather than individual parallel units. Such systems coordinate mitochondrial biogenesis, dynamics, and autophagic recycling with proteostasis to ensure the maintenance of high‐quality mitochondria and bioenergetically efficient cells. Neurons, post‐mitotic cells with high energy demands, depend heavily on these mechanisms and on the spatial coordination of MQC. Here, we discuss how failure of this integrated QC axis, rather than dysfunction of its individual components alone, can drive neuronal decline and contribute to the neurodegeneration.

## Graphical abstract

Quality control pathways form an integrated proteostasis–mitochondrial quality control axis that maintains mitochondrial and cellular homeostasis. Failure of this coordinated network drives neuronal decline and contributes to neurodegeneration.

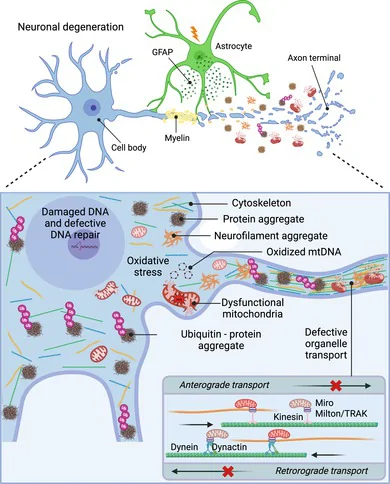

## 1 Introduction

Quality control (QC) processes are pivotal to the cell's economy, the balance between energy production and allocation of resources to preserve structural integrity, for optimizing cellular life cycle. Such processes prevent the accumulation of toxic damage by repairing, recycling, and/or eliminating defective components, including mitochondria (Ye et al. 2025).

QC systems are especially relevant in tissues that are mainly post‐mitotic for which housekeeping processes clear debris to support cell functions and tissue health (Panda et al. 2025). QC has long been conceptualized as a collection of discrete modules, including the proteostasis network (PN), which regulates proteome homeostasis, and mitochondrial quality control (MQC) pathways, which govern mitochondrial abundance and integrity (Misgeld and Schwarz 2017). However, increasing evidence indicates that QC systems operate through a hierarchically organized and spatially integrated regulatory axis rather than as a set of parallel, independent units. Together, QC systems coordinate mitochondrial biogenesis, dynamics, and autophagic recycling with proteostasis pathways to preserve mitochondrial integrity and sustain cellular bioenergetic competence.

Neurons are highly energy demanding cells whose viability relies heavily on efficient MQC. Mitochondrial biogenesis, which mainly occurs in the neuronal soma, generates new organelles some of which remain in the soma while others are transported to areas under high energy demands along axons and dendrites, such as presynaptic terminals and Ranvier nodes (Edgar et al. 2008; Misgeld et al. 2007). This intracellular distribution is guided by the microtubule and neurofilament network and relies on motor proteins, such as kinesins for anterograde and dyneins for retrograde transport (Hirokawa et al. 1991; Perkins et al. 2008; Sheng and Cai 2012; Susalka and Pfister 2000). Proteins from the mitochondrial Rho GTPase (Miro) family also play a role in regulating intra‐axonal movement of mitochondria (MacAskill et al. 2009). The transport of mitochondria and other cellular cargoes over the long distances in neurons, along with the maintenance of plasma membrane potential, is one of the greatest energetic demands in these cells. Because both processes rely heavily on mitochondrial ATP production, the preservation of high‐quality mitochondria is essential for neuronal function and survival (Sheng and Cai 2012).

Herein, we discuss how the failure of the QC integrated axis, rather than the dysfunction of its individual components, can trigger neuronal quality decline which is core to neurodegeneration pathophysiology.

## 2 Molecular Pathways of Integrated Proteostasis in Health and Aging

### 2.1 The Proteostasis Network

The PN is an adaptive system responsible for maintaining a metastable proteome via the coordination of chaperones, ubiquitin‐proteasome system (UPS), and autophagy–lysosome system. The PN operates via approximately 2000 factors in human cells and ensures that, upon synthesis, protein undergoes folding and achieves native conformation while preventing the generation and accumulation of dysfunctional and toxic aggregates (Klaips et al. 2018). Protein synthesis is the first critical node of the PN (Brandman and Hegde 2016). To avoid overwhelming the cellular folding capacity, bulk protein translation is tightly regulated and must be balanced with available folding resources (Brandman and Hegde 2016).

Accrual of misfolded proteins triggers endoplasmic reticulum (ER) stress and activates the unfolded protein response (UPR), via sensors like inositol‐requiring transmembrane kinase/endoribonuclease 1α (IRE1α), protein kinase RNA‐like endoplasmic reticulum kinase (PERK), and activating transcriptional factor (ATF) 6α (Ron and Walter 2007). Under basal conditions, these sensors are inactivated by their association to the ER chaperone binding‐immunoglobulin protein (BiP). Under stress, BiP dissociates, binds misfolded proteins, and triggers UPR activation (Bertolotti et al. 2000; Shen et al. 2002). PERK phosphorylates the eukaryotic initiation factor 2α (eIF2α). Phosphorylation of eIF2α is the central event of the integrated stress response (ISR), a conserved adaptive signaling network that transiently attenuates global protein synthesis while promoting the selective translation of stress‐responsive transcription factors, including ATF4. ATF4 translocates to the nucleus and induces the expression of chaperones and proteins involved in proteostasis recovery (Harding et al. 2001; Jousse et al. 2003). Furthermore, ATF6, processed in the Golgi apparatus, allows the release of a transcriptionally active factor that stimulates expression of chaperones and ER‐associated degradation components, whereas IRE1α promotes unconventional splicing of X‐box binding protein 1 (XBP1), generating XBP1s, a transcription factor that enhances ER proteostatic capacity (Walter and Ron 2011). Overall, the UPR alleviates protein load, increases folding capacity, and promotes clearance of damaged proteins.

**FIGURE 1:**
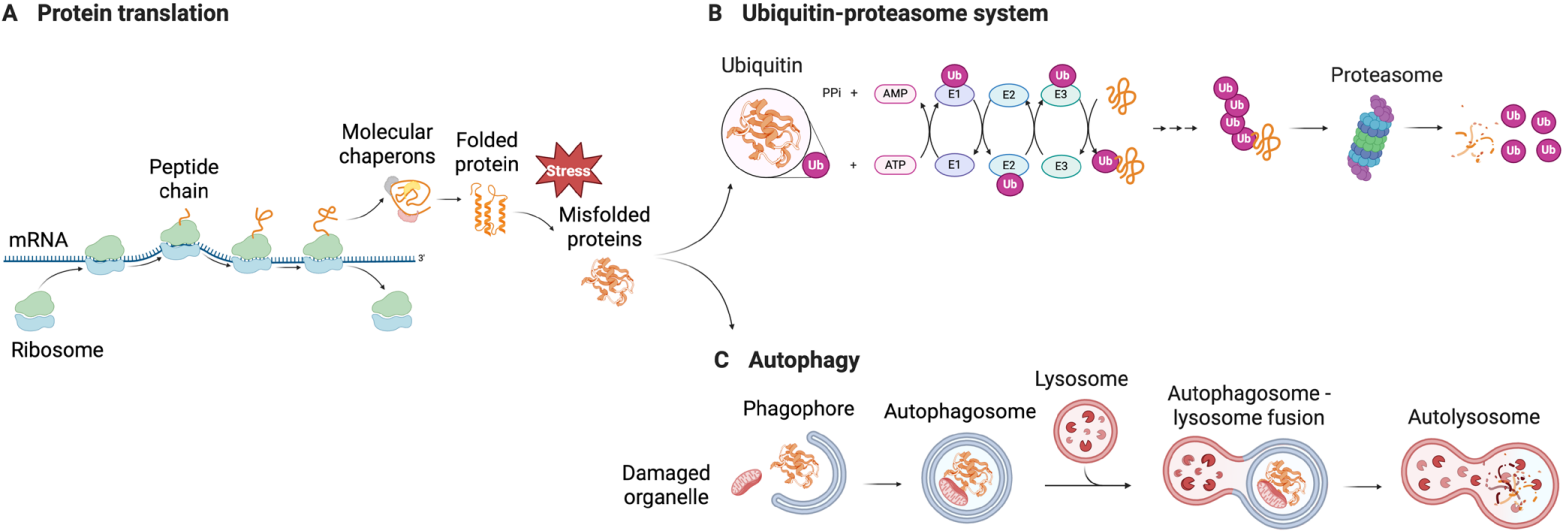
Cellular mechanisms of degradation of misfolded proteins. After protein translation (A), a stressful event can generate misfolded proteins. Cells can remove these deleterious proteins via the UPS (B), which involves ubiquitination by the E3 ligase followed by degradation by the proteasome, or through the autophagic pathway (C), involving the formation of an autophagosome that engulfs misfolded proteins and damaged organelles and fuses with the lysosome. Abbreviations: AMP, adenosine monophosphate; ATP, adenosine triphosphate; E1, ubiquitin‐activating enzyme E1; E2, ubiquitin‐conjugating enzyme; E3, E3 ubiquitin‐protein ligase; mRNA, messenger RNA; Ppi, inorganic pyrophosphate; Ub, ubiquitin; UPS, ubiquitin–proteasome system. Created with Biorender.com.

Polypeptide folding starts as soon as the protein emerges from the ribosome and represents an inherently error‐prone process whereby nascent chains must sequester hydrophobic residues to reach a low free‐energy native state (Bartlett and Radford 2009). Folding occurs vectorially on the ribosome, where cotranslational folding mechanisms limit the exposure of vulnerable non‐native states. In the crowded cellular environment, partially folded intermediates are at high risk of forming aberrant intermolecular contacts, leading to formation of amorphous aggregates and structured amyloid fibrils (Klaips et al. 2018) (Figure 1A).

Molecular chaperones are facilitators of the PN, acting at every stage from de novo folding to degradation (Brehme et al. 2014; Sala et al. 2017). The human “chaperome” is composed of approximately 330 members, including the heat shock protein (Hsp) 70 and Hsp90 families, which prevent aggregation by shielding hydrophobic domains and favoring the folding landscape (Brehme et al. 2014; Sala et al. 2017). Chaperone functions are involved in a complex and multi‐stage proteostasis system that extends beyond cells' confines to encompass extracellular and systemic environments (Gomes and Vendruscolo 2026).

Terminally misfolded or damaged proteins must be efficiently cleared to prevent proteotoxicity. The PN utilizes two major degradation components: (1) the UPS comprising about 850 components that primarily targets individual, soluble misfolded proteins for degradation to the 26S proteasome (Figure 1B); and (2) the autophagy‐lysosome system that includes about 500 components and is responsible for clearing larger protein aggregates and damaged organelles (Xie and Klionsky 2007; Korolchuk et al. 2010) (Figure 1C).

Before degradation, aggregated proteins can undergo chaperone‐mediated disaggregation. The Hsp70–Hsp40–Hsp110 triad represents the core of the metazoan disaggregation machinery. This system can solubilize amyloid‐like aggregates, a process that is critically dependent on the nucleotide exchange factor Hsp110 (Nillegoda and Bukau 2015). Proteins released from aggregates may subsequently undergo chaperone‐assisted refolding or, when recovery is not possible, be directed toward proteolytic clearance, including degradation by the UPS. The UPS supervises the bulk of cytosolic protein degradation. Ubiquitin (Ub) is a 76‐amino acid polypeptide that, in monomeric or polymeric form, is attached to proteins by a set of ubiquitin enzymes (E1, E2, and E3) (Kleiger and Mayor 2014). Specifically, using ATP, E1 and E2 conjugate Ub to substrates, while E3 catalyzes the binding of the C‐terminal carboxyl group of Ub to the target protein. In mammals, Parkin is one of E3 ligases involved in conferring specificity to the recognition of substrates destined to the 26S proteasome for degradation (Aguileta et al. 2015). Proteasomal degradation of the target protein requires Lys48‐type polyubiquitination. The ubiquitin‐conjugating enzyme E2 O (UBE2O), an atypical E2/E3 hybrid ubiquitin ligase, selectively targets “orphan” proteins, including unassembled subunits that fail to incorporate into their native protein complexes, thereby promoting their ubiquitination and proteasomal degradation (Yanagitani et al. 2017). This mechanism is vital for avoiding the accumulation of hydrophobic surfaces that seed aggregation. Alternatively, misfolded proteins and/or damaged organelles can be cleared via the autophagy pathway operating through formation of an autophagosome which fuses with lysosomes for degradation (Picca et al. 2023).

### 2.2 The Proteostasis Network in Aging and Neurodegeneration

Aging is characterized by a decline in proteostasis, particularly in neurons (Guldner et al. 2026; Shrestha et al. 2026). Disruption of the Hsp70–Hsp40–Hsp110 triad stoichiometry is one relevant alteration in PN leading to a proteostatic collapse where metastable proteins, many of which are essential for synaptic signaling, are sequestered into toxic aggregates (Klaips et al. 2018). Moreover, high‐molecular‐weight protein aggregates can directly impair proteasome function or saturate its degradative capacity, thereby compromising the clearance of orphan proteins and other misfolded substrates and promoting the progressive proteostasis collapse characteristic of neurodegeneration (Borlepawar et al. 2026).

A dynamic interplay between neuronal proteostasis and microglial function has been described in the context of aging and neurodegeneration (Guldner et al. 2026). Age‐related loss of neuronal proteome maintenance and increase of neuronal protein half‐life was described in aged mice (Guldner et al. 2026). Moreover, a neuronal “aggregome” consisting of more than 1700 proteins, with about half showing age‐associated reduced degradation, has been reported (Guldner et al. 2026). Of note, neuronal synaptic proteins are enriched in aged microglia, indicating a link between impaired synaptic protein turnover and aggregation to microglial engulfment of synapses, ultimately contributing to synapse degeneration and loss and cognitive decline during aging (Guldner et al. 2026). These findings highlight the critical role of microglial proteostasis in neuronal health and disease progression.

Further to this, systems‐level organization of extracellular proteostasis has been described (Gomes and Vendruscolo 2026). This network would operate across pericellular, tissue, and systemic organization levels, whereby several secreted factors, including chaperones and proteases, cooperate to recognize and remove misfolded proteins. Failures in such hierarchical defense mechanisms are pivotal for developing neurodegenerative disorders and systemic amyloidosis (Gomes and Vendruscolo 2026).

The concept of proteostasis has thus evolved from a purely intracellular phenomenon to a broader, integrated systems‐level network relevant for organismal health. The complexity of neurodegenerative diseases and their molecular heterogeneity highlight the need for a deeper understanding of protein aggregation patterns. A Pan‐neurodegeneration Atlas (PanNDA), a large‐scale multilayer proteomic analysis of human brain samples across six major neurodegenerative diseases (i.e., Alzheimer's disease, frontotemporal lobar degeneration with TDP‐43 pathology, Lewy body dementia, Parkinson's disease, progressive supranuclear palsy with tau pathology, and vascular dementia), has been generated (Shrestha et al. 2026). This atlas integrates data from whole proteome, detergent‐insoluble proteome (representing aggregated proteins), and post‐translational modifications, revealing distinct molecular subtypes within neurodegenerative conditions and identifying shared alterations and hub regulators within protein networks. These proteomic insights are instrumental for elucidating specific protein QC pathways affected in different neurodegenerative diseases and developing targeted therapeutic interventions (Shrestha et al. 2026).

Altogether these data indicate that while the understanding of proteostasis and the role of molecular chaperones in preventing protein misfolding and aggregation remains crucial (Klaips et al. 2018), understanding their network is also very relevant. To fully capture this, a shift toward a more integrated view, including the dynamic interplay between neuronal and microglial proteostasis, the hierarchical organization of extracellular proteostasis, and the characterization of protein aggregation in neurodegenerative diseases is needed (Gomes and Vendruscolo 2026).

## 3 Mitochondria and Proteostasis

### 3.1 Organization of the Mitochondrial Proteome and Mitochondrial Proteostasis

Mitochondria derive from an endosymbiotic progenitor traceable to *Alphaproteobacteria* (Zimorski et al. 2014). Their bacterial origin is supported by several structural features such as a circular mitochondrial DNA (mtDNA), small mitoribosomes, cardiolipin, as well as a double membrane system of an outer membrane (OMM) and an inner membrane (IMM) enclosing the intermembrane space (IMS). The cristae of the IMM increase the surface area for allocating the electron transport chain (ETC) complexes (Roger et al. 2017), while its low permeability maintains the electrochemical gradient that is fundamental for ATP synthesis via chemiosmosis. The OMM, instead, is highly permeable due to several voltage‐gated anion channels (VDACs) allowing the transit of solutes up to 5 kDa (Gonçalves et al. 2007).

**FIGURE 2:**
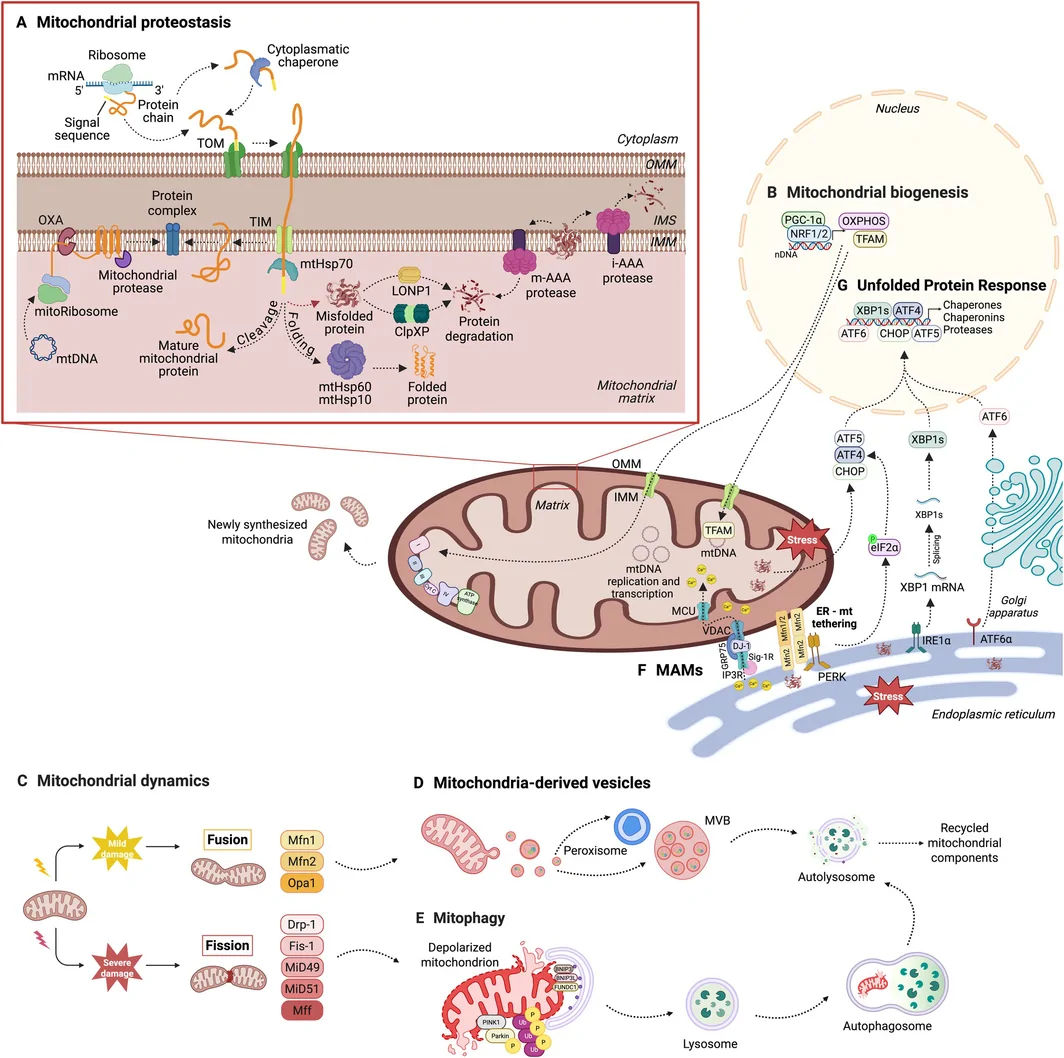
Representation of the molecular pathways linking mitochondrial proteostasis to mitochondrial quality control processes. (A) Mitochondrial protein import is mediated by the TOM and TIM translocase complexes. Misfolded or damaged proteins within the mitochondrial matrix and IMM are refolded by molecular chaperones, including mtHsp70, mtHsp60, and mtHsp10, or degraded by ATP‐dependent proteases such as LONP1 and ClpXP. The m‐AAA and i‐AAA proteases further support the elimination of oxidized or misassembled proteins, adapting their activity to mitochondrial stress. (B) Mitochondrial biogenesis, driven by the PGC1α–NRF1/2–TFAM signaling axis, generates new functional mitochondria that integrate into the mitochondrial network. (C) Mitochondrial network homeostasis is maintained through the balanced processes of fusion and fission dynamics. Fusion is mediated by Mfn1, Mfn2, and OPA1, whereas fission is driven by DRP1, which is recruited to the OMM by FIS1, MFF, and MiD49/51. (D) Mildly damaged mitochondria can release MDVs, containing mitochondrial components, which can be directed to peroxisomes or endolysosomes for degradation. (E) When mitochondrial dysfunction becomes severe, depolarized and bioenergetically impaired mitochondria are eliminated by mitophagy through PINK1–Parkin‐dependent or ‐independent pathways that promote autophagosome formation and lysosomal degradation. (F) Mitochondria are physically connected to the ER through MAMs, which coordinate Ca^2+^ and lipid transfer to regulate mitochondrial metabolism, dynamics, autophagy, apoptosis, and the ER stress response. Ca^2+^ exchange is primarily mediated by the IP3R–Grp75–VDAC complex, with additional contributions from DJ‐1, MCU, Sig‐1R, and Mfn1/2. (G) Accumulation of unfolded or misfolded proteins in mitochondria and/or the ER activates the mitochondrial and endoplasmic reticulum unfolded protein responses (UPR^mt^ and UPR^ER^), enhancing proteostasis through increased expression of molecular chaperones, chaperonins, and proteases. UPR^mt^ primarily involves the transcription factors ATF5, ATF4, and CHOP, whereas UPR^ER^ is mediated through the PERK–eIF2α, IRE1α–XBP1s, and ATF6 signaling pathways. Abbreviations: ATF, activating transcription factor; BNIP3, BCL2/adenovirus E1B 19‐kDa protein‐interacting protein 3; BNIP3L, BNIP3 like; Ca^2+^, calcium ion; CHOP, C/EBP homologous protein; ClpXP, ATP‐dependent Clp protease proteolytic complex; CytC, cytochrome C; DJ‐1, protein/nucleic acid deglycase DJ‐1; Drp‐1, dynamin‐related protein‐1; eIF2α, eukaryotic initiation factor 2α; ER, endoplasmic reticulum; Fis‐1, fission 1 protein; FUNDC1, FUN14 domain‐containing 1; Grp75, glucose‐regulated protein 75; IMM, inner mitochondrial membrane; IMS, intermembrane space; IP3R, inositol‐1,4,5‐trisphosphate receptor; IRE1α, inositol‐requiring transmembrane kinase/endoribonuclease 1α; LONP1, Lon protease 1; MAMs, mitochondrial‐associated membranes; MCU, mitochondrial calcium uniporter; MDV, mitochondria‐derived vesicle; Mff, mitochondrial fission factor; Mfn1/2, mitofusin‐1/2; MiD49/51, mitochondrial dynamics proteins 49 and 51 kDa; MQC, mitochondrial quality control; mtDNA, mitochondrial DNA; mtHsp70/60/10, mitochondrial heat shock protein 70/60/10; MVB, multivesicular body; NRF1/2, nuclear respiratory factor 1/2; OMM, outer mitochondrial membrane; Opa1, optic atrophy protein 1; OXA, oxidase assembly translocase; OXPHOS, oxidative phosphorylation; P, phosphate residue; PERK, protein kinase R‐like endoplasmic reticulum kinase; PGC1α, peroxisome proliferator‐activated receptor‐γ coactivator‐1α; PINK1, PTEN‐induced kinase‐1; Sig‐1R, sigma‐1 receptor; TFAM, mitochondrial transcription factor A; TIM23, translocase of the inner membrane 23; TOM, translocase of the outer membrane; Ub, ubiquitin, VDAC, voltage‐dependent anion channels, XBP1s, splicing of X‐box binding protein 1. Created with BioRender.com.

The mitochondrial proteome consists of thousands of proteins in both yeast and humans (Morgenstern et al. 2017), of which only 13 are encoded autonomously by the mtDNA. Most proteins are nuclear‐encoded, synthesized in the cytosol, and imported into mitochondria in an unfolded form through a complex network of protein translocases of the OMM (TOM) and IMM (TIM), in a process dependent on ATP, matrix metalloproteinases (MMPs), and mitochondrial chaperones such as mtHsp70 (Moulin et al. 2019) (Figure 2A). Once in the matrix, tagging sequences are removed and mature proteins are sorted by additional chaperones (Endo et al. 2011; Pfanner et al. 2019). Coordinated expression of nuclear and mitochondrial genomes is essential to avoid the accumulation of unassembled subunits, which is associated with increased production of reactive oxygen species (ROS) (Endo et al. 2011; Ott et al. 2016; Priesnitz and Becker 2018; Suhm et al. 2018). Beyond energy metabolism, mitochondria contribute to lipid and amino acid biosynthesis, urea cycle, heme and iron–sulfur clusters synthesis, apoptosis, and innate immunity. They also communicate with other organelles, such as the ER, via membrane contact sites to promote lipid exchange and calcium signaling (Pfanner et al. 2019; Prinz et al. 2020).

Mitochondrial integrity is preserved by the coordinated activity of cytosolic QC systems, such as the UPS, and intramitochondrial processes (Ruan et al. 2020). Within the matrix, chaperones promote proper folding and limit aggregation of both nuclear‐ and mitochondrial‐encoded proteins. Ubiquitinated substrates are recognized by recruitment factors (Heo et al. 2010; Wu et al. 2016), which engage AAA‐ATPase proteases, such as the ATP‐dependent protein dislocase merozoite surface protein 1 (Msp1) (Chen et al. 2014; Okreglak and Walter 2014) and the cell division cycle 48 (Cdc48)‐associated mitochondrial stress‐responsive 1 (Vms1) (Heo et al. 2010) in yeast and the valosin‐containing protein (VCP) in mammals (Kim et al. 2013; Xu et al. 2011). These complexes extract proteins from the OMM using ATP hydrolysis and target them for degradation by cytosolic proteasomal complex (Chen et al. 2014; Wu et al. 2016). A ribosome quality control system also contributes to mitochondrial proteostasis (Izawa et al. 2017).

Polypeptides undergoing translation arrest are recognized by the E3 ubiquitin ligase Ltn1 (Listerin in mammals), which ubiquitinates incomplete translation products and targets them for proteasomal degradation. In parallel, ribosome quality control complex subunit 2 (Rqc2) catalyzes the addition of C‐terminal alanine/threonine‐rich extensions (CAT tails) to stalled polypeptides. When Ltn1‐mediated ubiquitination is impaired or overwhelmed, CAT‐tailed proteins can escape degradation, enter mitochondria, and accumulate within the matrix, where they promote proteotoxic stress and protein aggregation (Izawa et al. 2017; Zurita Rendón et al. 2018). Although the precise mechanism remains unclear, Vms1 counteracts this by interfering with Rqc2‐mediated CAT tailing and likely acting as a tRNA hydrolase to release stalled polypeptides from 60S ribosomes (Izawa et al. 2017; Zurita Rendón et al. 2018). The accumulation of misfolded proteins within mitochondria activates the mitochondrial UPR (UPRmt), which induces the expression of nuclear genes involved in proteostasis (Moehle et al. 2019; Shpilka and Haynes 2018) and transiently reduces protein import, possibly due to saturation of mtHsp70 (Lim et al. 2001). This leads to cytosolic accumulation of precursors and activation of heat shock transcription factor 1 (HSF1) and 26S proteasome regulatory subunit repetitive‐element protein 4, increasing the expression of chaperones and proteasome components (Boos et al. 2019) and promoting degradation of mistargeted proteins (Wrobel et al. 2015). In parallel, the linker protein Cis1 recruits Msp1 to remove proteins stalled in import channels (mitoCPR) (Weidberg and Amon 2018), while UBX domain‐containing protein 2 (Ubx2) cooperates with the TOM complex and Cdc48 through mitochondrial protein translocation‐associated degradation pathway (mitoTAD) (Mårtensson et al. 2019). Precursor stress reduces cytosolic translation, alleviating proteotoxic burden (Wang and Chen 2015). However, how these pathways are coordinated and how UPR‐induced proteins escape cytosolic degradation remain unclear. Overall, cytosolic protein QC pathways, primarily UPS, and intramitochondrial QC mechanisms, including mitochondrial chaperones, proteases, and the UPRmt, are tightly interconnected. Cytosolic pathways preserve mitochondrial proteostasis, while mitochondrial stress responses contribute to the maintenance of cytosolic proteostasis. The major pathways contributing to mitochondrial proteostasis and their integration with broader MQC mechanisms are summarized in Table 1.

**TABLE 1:** Pathways of proteostasis and integrated mitochondrial quality control mechanisms.

QC pathway/mitochondrial proteostasis	Main role	Molecules involved	Role in mitochondrial proteostasis	References
Mitochondrial protein import (TOM/TIM pathway)	Transport of nuclear‐encoded proteins into the mitochondrion	TOM complex (TOM20, TOM40, TOM70), TIM complex, mtHsp70, cytosolic chaperones, ATP, MMPs	Prevents accumulation of cytosolic protein precursors; ensures correct assembly of the mitochondrial proteome; defects induce proteotoxic stress	(Endo et al. 2011; Moulin et al. 2019; Pfanner et al. 2019)
Mitochondrial folding chaperones	Correct folding of imported and mitochondrial proteins	mtHsp70/HSPA9, Hsp60, Hsp10, Hsp40, HspD1, HspE1	Maintain proteins in a functional state, prevent aggregation and facilitate assembly of OXPHOS complexes	(Endo et al. 2011; Münch 2018)
UPS associated with mitochondria	Elimination of defective mitochondrial proteins exposed on the cytosolic side	E3 ligases, 26S proteasome, VCP/p97, Cdc48, MARCH5/MITOL, MULAN, Mdm30, Parkin, ubiquitin	Removes damaged or mis‐assembled OMM proteins; maintains OMM proteostasis; modulates stability of Drp‐1 and mitofusins; connects dynamics and proteostasis	(Escobar‐Henriques et al. 2006; Karbowski et al. 2007; Livnat‐Levanon and Glickman 2011; Ruan et al. 2020)
AAA‐ATPases	Removal of aberrant proteins from the OMM	Msp1 (AAA‐ATPase), Vms1, Cdc48, VCP/p97	Extract stalled or improperly inserted proteins from the outer mitochondrial membrane for proteasomal degradation	(Chen et al. 2014; Heo et al. 2010; Kim et al. 2013; Okreglak and Walter 2014)
MitoCPR	Removal of proteins stalled in import channels	Cis1, Msp1, TOM complex	Clears TOM channels occupied by mis‐imported precursors, preventing saturation of the import system	(Weidberg and Amon 2018)
MitoTAD	Degradation of non‐imported mitochondrial precursors	Ubx2, TOM complex, Cdc48/p97, ubiquitin	Eliminates cytosolic proteins addressed to the mitochondrion but stalled during translocation	(Mårtensson et al. 2019)
RQC	Quality control of arrested translation	Rqc2, CAT tails, E3 ligases, Vms1	Prevents entry into the mitochondrion of truncated and toxic polypeptides derived from stalled ribosomes	(Izawa et al. 2017; Zurita Rendón et al. 2018)
UPRmt	Retrograde mitochondrion‐to‐nucleus response to proteotoxic stress	ATFS‐1 ( *C. elegans* ), ATF5 (mammals), ATF4, HSF1, CHOP, LONP1, HspD1, HspE1, HspA9	Induces chaperones, proteases and proteostatic recovery programs; reduces import when the system is overloaded	(Fiorese et al. 2016; Moehle et al. 2019; Nargund et al. 2015; Shpilka and Haynes 2018)
Mitochondrial matrix proteases	Selective degradation of damaged or aggregated proteins	LONP1, CLPP/CLPX, Oma1, m‐AAA protease, i‐AAA protease	Remove oxidized, misfolded or unassembled proteins; regulate protein turnover	(Haynes et al. 2007; Münch 2018)
MAGIC	Transfer of cytosolic aggregates toward the mitochondrion	Hsp104, LONP, mitochondrial import	Disposal mechanism for cytosolic aggregates; links cytoplasmic and mitochondrial proteostasis	(Ruan et al. 2017; Shorter and Southworth 2019)
Mitochondrial biogenesis	Production of new mitochondria and renewal of the mitochondrial proteome	PGC‐1α/β, NRF1, NRF2, TFAM, mtDNA	Coordinates nuclear and mitochondrial protein synthesis, avoiding accumulation of unassembled OXPHOS subunits	(Bonawitz et al. 2006; Scarpulla et al. 2012)
Mitochondrial fission	Separation of damaged segments of the mitochondrial network	Drp‐1, Mff, Fis‐1, MiD49, MiD51	Isolates depolarized or ROS‐rich mitochondria, facilitating mitophagy and turnover; protects mitochondria from pearling	(Smirnova et al. 2001; Sturm et al. 2025; Twig et al. 2008)
Mitochondrial fusion	Functional complementation between mitochondria	Mfn1, Mfn2, Opa1	Dilutes damage through the exchange of mtDNA, metabolites and proteins; preserves protein homeostasis	(Olichon et al. 2003; van der Bliek et al. 2013)
Mfn2 as proteostatic sensor	Link between dynamics and quality control	Mfn2, HSC70, Hsp90, proteasome	Recruits cytosolic chaperones (HSC70 and Hsp90) and proteasome; limits protein aggregation	(Joaquim and Escobar‐Henriques 2020; Joaquim et al. 2025)
Ubiquitin‐dependent mitophagy	Elimination of irreversibly damaged mitochondria	PINK1, Parkin, ubiquitin, p62/SQSTM1, OPTN	Removes depolarized organelles, and with elevated ROS or mtDNA damage	(Fang et al. 2019; Onishi et al. 2021)
Ubiquitin‐independent receptor‐mediated mitophagy	Selective removal of damaged mitochondria	BNIP3, NIX/BNIP3L, FUNDC1	Activates mitochondrial autophagy independently of ubiquitination	(Kagan et al. 2016; Picca et al. 2023)
Cardiolipin‐mediated mitophagy	Lipid signal for mitochondrial elimination	Cardiolipin, LC3	Converts mitochondrial lipids into autophagic recruitment signals	(Sentelle et al. 2012)
MDVs	Selective elimination of damaged mitochondrial components	MDVs, Parkin, lysosomes	Allows removal of specific cargo without degradation of the entire organelle	(Neuspiel et al. 2008; Soubannier et al. 2012)
MMEL	Local remodeling of mitochondria under stress	Lysosomes incorporated into megamitochondria	New QC modality that preserves the organelle while maintaining local degradative activity	(Hao et al. 2023)
MAMs	ER‐mitochondrion communication for metabolism and stress	IP3R, VDAC, Grp75, Mfn2, Sigma‐1 receptor, MCU	Regulates Ca^2+^, protein folding, autophagy and ER stress response	(Hayashi and Su 2007; Vance 2014)
ER stress–mitochondrial proteostasis axis	Coordination between ER‐UPR and mitochondrial function	PERK, ATF4, CHOP, ERO1α	Modulates ROS, metabolism and apoptosis; links ER and mitochondrial proteostasis	(Harding et al. 2003; Puthalakath et al. 2007)
MAM‐mediated autophagosome formation	Formation of autophagosomes at ER‐mitochondrion sites	ULK1, ATG14, ATG2, WIPI1, Beclin‐1, AMBRA1, STX17	Integrates stress, autophagy and mitochondrial clearance	(Hamasaki et al. 2013; Antonioli et al. 2014)
Mitokine signaling	Systemic communication of mitochondrial stress	Mitokines, neuronal signals, peripheral UPRmt	Coordinates the proteostatic response between different tissues	(Zhang et al. 2021; Berendzen et al. 2016)

Abbreviations: AAA‐ATPases, ATPases associated with diverse cellular activities; AMBRA1, activating molecule in BECN1‐regulated autophagy protein 1; ATF, activating transcription factor; ATFS, activating transcription factor associated with stress‐1; ATG, autophagy‐related protein; ATP, adenosine triphosphate; BNIP3, BCL2 interacting protein 3; Ca^2+^, calcium ion; CAT tails, carboxy‐terminal alanine and threonine tails; Cdc48, cell division cycle protein 48; CHOP, C/EBP homologous protein; Cis1, cytokine‐inducible SH2‐containing protein; CLPP, caseinolytic mitochondrial matrix peptidase proteolytic subunit; CLPX, caseinolytic mitochondrial matrix peptidase chaperone subunit X; Drp‐1, dynamin‐related protein 1; ER, endoplasmic reticulum; ERO1α, endoplasmic reticulum oxidoreductin‐1 alpha; Fis‐1, mitochondrial fission 1 protein; FUNDC1, FUN14 domain‐containing protein 1; Grp75, glucose‐regulated protein 75; HSC70, heat shock cognate 71 kDa protein; HSF, heat shock factor; Hsp, heat shock protein; i‐AAA protease, intermembrane‐space‐exposed ATPases associated with diverse cellular activities; IP3R, inositol 1,4,5‐trisphosphate receptor; LC3, microtubule‐associated protein 1 light chain 3; LOMP, Lon protease homolog, mitochondrial; m‐AAA protease, matrix‐side‐oriented ATPases associated with diverse cellular activities; MAGIC, mitochondria as guardian in cytosol; MAM, ER–mitochondria contact sites; MARCH5/MITOL, membrane‐associated ring‐ch‐type finger 5/mitochondrial ubiquitin ligase; MCU, mitochondrial calcium uniporter; Mdm, mitochondrial distribution and morphology protein; Mff, mitochondrial fission factor; MDV, mitochondria‐derived vesicle; mitoCPR, mitochondrial import stress QC; Mfn1/2, mitofusin; MiD49/51, mitochondrial dynamics protein MID49/51; mitoTAD, mitochondrial protein translocation‐associated degradation; MMEL, megamitochondria engulfing lysosomes; MMP, matrix metalloproteinases; Msp1, merozoite surface protein 1; mtDNA, mitochondrial DNA; mtHsp, mitochondrial heat shock protein; MULAN, mitochondrial ubiquitin ligase activator of NF‐κB; NIX/BNIP3L, BCL2 interacting protein 3 like; NRF1/2, nuclear respiratory factor 1; Oma1, metalloendopeptidase OMA1, mitochondrial; OMM, outer mitochondrial membrane; Opa1, optic atrophy protein 1; OPTN, optineurin; OXPHOS, oxidative phosphorylation; p62/SQSTM1, ubiquitin‐binding protein p62/sequestosome‐1; PERK, protein kinase R (PKR)‐like endoplasmic reticulum kinase; PGC‐1α/β, peroxisome proliferator‐activated receptor gamma coactivator 1‐alpha/beta; PINK1, PTEN‐induced kinase 1; QC, quality control; ROS, reactive oxygen species; RQC, ribosome‐associated quality control; Rqc2, ribosome quality control complex subunit 2; STX17, syntaxin‐17; TFAM, Transcription factor A, mitochondrial; TIM, translocase of the inner membrane; TOM, translocase of the outer membrane; Ubx2, ubiquitin‐regulatory X domain‐containing protein 2; ULK1, unc‐51 like autophagy activating kinase 1; UPRmt, mitochondrial unfolded protein response; VCP/p97, valosin‐containing protein; VDAC, voltage‐dependent anion channel; Vms1, VCP/Cdc48‐associated mitochondrial stress‐responsive 1; WIPI1, WD repeat domain phosphoinositide‐interacting protein 1.

Mitochondria act as sensors and buffers of proteostatic stress. Acute stress or aging promote protein aggregation (Hipp et al. 2019), often originating in the ER and accumulating at ER–mitochondria contact sites (Zhou et al. 2014). In yeast, these aggregates are retained in the mother cell and cleared through the mitochondria as guardian in cytosol (MAGIC) pathway (Ruan et al. 2017), which requires Hsp104 to disaggregate proteins that are then imported into mitochondria (Shorter and Southworth 2019). Once internalized, misfolded proteins are degraded by mitochondrial proteases such as the Lon protease (LONP). Even under physiological conditions, structurally unstable cytosolic proteins can be constitutively targeted to mitochondria for degradation (Ruan et al. 2017) and defects in ER targeting (e.g., signal recognition particle, SRP, dysfunction) lead to their mitochondrial accumulation. This process is conserved in human cells: misfolded proteins have been shown to be imported in human retinal pigment epithelial cells (Ruan et al. 2017), while in HeLa cells, FUN14 domain‐containing protein 1 (FUNDC1) and Heat Shock Cognate 70 kDa protein (HSC70) facilitates their import and degradation by Lon protease 1 (LONP1) (Li et al. 2019). Therefore, the MAGIC pathway may be an evolutionarily conserved mechanism for maintaining cytosolic proteostasis. Defects in mitochondrial protein import have been associated with cytosolic accumulation of immature and aberrant protein precursors, overwhelming proteostasis mechanisms and facilitating the misfolding and pathological aggregation of proteins such as α‐synuclein and β‐amyloid (Nowicka et al. 2021). This process disrupts cytosolic proteostasis and triggers chaperone‐mediated stress responses contributing to neurodegeneration (Nowicka et al. 2021).

### 3.2 Mitochondrial Quality Control System

Mitochondria establish a dynamic, constantly remodeled network through the integration of MQC processes, including biogenesis, fusion and fission (dynamics), proteostasis, and mitophagy (Giacomello et al. 2020), whose major components and roles are summarized in Table 1. MQC contributes to mitostasis, the long‐term metabolic adaptation and maintenance of the mitochondrial population. In particular, the coordinated balance between biogenesis and mitophagy ensures continuous renewal of organelles (Panda et al. 2025; Ploumi et al. 2017).

Mitochondrial biogenesis, triggered by energy demands and/or metabolic stress, is mainly regulated by the nucleus via the proliferator‐activated receptor gamma coactivators 1 (PGC1α and PGC1β) which induce the expression of the mitochondrial transcription factor A (TFAM), via the nuclear respiratory factors (NRF1 and NRF2) (Scarpulla et al. 2012). TFAM, in the mitochondrion, is responsible for activating mtDNA transcription and replication and consequently the generation of new organelles (Bonawitz et al. 2006; Picca and Lezza 2015) (Figure 2B). The biogenesis process also involves the synthesis of nuclear‐encoded mitochondrial proteins and their subsequent import into mitochondria. Because newly synthesized proteins are prone to aggregation and can induce cytosolic proteostatic stress, a tight coordination between transcription and protein import is essential. In budding yeast, TOM70 plays an evolutionarily conserved transcriptional regulatory role, coordinating biogenesis and import to maintain cytosolic proteostasis (Liu et al. 2022). Furthermore, the aging‐associated decline in TOM70 contributes to mitochondrial dysfunction, while its overexpression attenuates mitochondrial defects and prolongs replicative lifespan (Liu et al. 2022).

Mitophagy selectively removes dysfunctional mitochondria with irreversible damage via autophagocytosis and transport to lysosomes for degradation (Figure 2E). This process can be triggered by several stressors, including damage to mtDNA, mitochondrial membrane depolarization, altered mitochondrial fission–fusion dynamics, high ROS production, and increased oxidative stress (Picca et al. 2023). Depending on the receptors and/or ubiquitin‐autophagy adaptors recruited, mitophagy can be classified as ubiquitin‐dependent (including PARKIN‐dependent and PARKIN‐independent pathways), receptor‐mediated ubiquitin‐independent, lipid‐ (or cardiolipin‐) mediated, sphingolipid‐mediated, or micromitophagy (Sentelle et al. 2012; Lemasters 2014; Dany et al. 2016; Kagan et al. 2016; Picca et al. 2023). Mitophagy also plays relevant roles during mammalian development (Palikaras et al. 2018) and, after fertilization, cooperates with UPS in the selective elimination of paternal mitochondria (Song et al. 2016). Impaired mitophagy leads to accumulation of defective organelles and contributes to several disease conditions, including neurodegeneration (Fang et al. 2019; Onishi et al. 2021; Picca et al. 2023). Disrupted mitostasis is also associated with the accumulation of ROS and reactive nitrogen species (RNS) damage to mitochondria and may lead to the release of organellar components into the cytosol, triggering an inflammatory response. In this setting, cell death may occur, thus resulting in a heavy perturbation of metabolism and cellular signaling mechanisms (Kong et al. 2025; Picca et al. 2021; Vanportfliet et al. 2024).

Due to the high oxygen and glucose demand, the brain is one of the most vulnerable tissues to mitochondrial dysfunction. The loss of mitochondrial integrity, indeed, is closely linked to aging and neurodegeneration (Lionaki et al. 2015; Panda et al. 2025). Mitochondrial dysfunction and metabolic alterations contribute to both the onset and progression of these conditions. At the same time, neurodegenerative processes, such as the formation of amyloid plaques and altered tau protein, further impinge on mitochondrial homeostasis (Khan et al. 2024; Klemmensen et al. 2024; Marzetti et al. 2025), establishing a vicious circle in which mitochondrial damage and neurodegeneration mutually reinforce each other.

#### 3.2.1 Mitochondrial Dynamics: At the Interface of MQC and Proteostasis

Mitochondria exist in a highly dynamic network continuously remodeled via fusion, fission, and transport along microtubules (Braschi and McBride 2010). The balance between these processes is also a QC filter for preserving mitostasis. Fission segregates damaged mitochondrial portions, with low membrane potential and high ROS, from the healthy network (Twig et al. 2008), while fusion dilutes damage via exchange of mtDNA, metabolites, and proteins (Olichon et al. 2003). mtDNA copies are organized into nucleoids distributed along the mitochondrial network and linked to IMM cristae, supporting both inheritance during cell division and homogeneous transcriptional activity (Iborra et al. 2004; Jajoo et al. 2016; Jakubke et al. 2021; Nunnari et al. 1997). However, the mechanisms governing intramitochondrial nucleoid trafficking remain poorly understood (Macuada et al. 2025).

Key regulators of mitochondrial dynamics include dynamin‐related protein 1 (Drp‐1) and its adaptors, such as mitochondrial fission factor (Mff), fission protein 1 (Fis‐1), and mitochondrial dynamics proteins MiD49 and MiD51, which, under conditions of mitochondrial stress, recruit Drp‐1 to fission sites. The GTPase Drp‐1 mediates membrane constriction and scission (Ingerman et al. 2005; Losón et al. 2013; Mozdy et al. 2000; Smirnova et al. 2001), a prerequisite for selective mitophagy. Conversely, fusion, orchestrated by the GTPases mitofusin‐1 and mitofusin‐2 (Mfn1/2), located at the OMM, and optic atrophy 1 (Opa1) on the IMM, counteracts mild stress by promoting the exchange between partially damaged and healthy mitochondria (van der Bliek et al. 2013) (Figure 2C).

Mitochondria are also integrated into cellular signaling networks through physical contacts with organelles such as the ER, plasma membrane, and peroxisomes (Klecker et al. 2014; Westermann 2015). Mitochondrial‐associated membranes (MAMs), specialized mitochondria–ER contact sites, regulate mitochondrial biogenesis, Ca^2+^ homeostasis, lipid metabolism, and apoptosis (Daniele and Schiaffino 2014; Kornmann et al. 2009). Recently, a reversible morphological transition state termed mitochondrial pearling has been described in both yeast *Saccharomyces cerevisiae* and human cells in which mitochondria adopt a “beads‐on‐a‐string” morphology while preserving membrane and matrix continuity (Sturm et al. 2025). Initially associated with stress or alterations in dynamics (Cho et al. 2017; Legesse‐Miller et al. 2003), pearling is now considered a rapid, evolutionarily conserved physiological response (Sturm et al. 2025). Contacts with the ER are crucial for triggering this process for which mitochondrial calcium uniporter (MCU) and inverted formin‐2 proteins are needed (Cho et al. 2017), while cristae organization modulates its frequency and persistence (Stephan et al. 2020). Cells lacking Drp‐1 display increased pearling likely due to loss of compartmentalization and increased mechanical tension in hyperfused networks, which promotes propagation of morphological instability (Sturm et al. 2025). Thus, fission may have an indirect protective role. Mitochondrial morphology and distribution also influence cellular physiology. Perinuclear accumulation of mitochondria can increase oxidative stress and modulate hypoxia‐responsive gene expression (Al‐Mehdi et al. 2012). This structural plasticity allows rapid adaptation to environmental and intracellular changes, contributing to stress management and cell fate regulation (Cheng et al. 2023).

Mitochondrial dynamics is closely linked to cytosolic proteostasis pathways, particularly the UPS, which regulates the turnover and activity of OMM proteins involved in fusion and fission during proteotoxic stress. OMM proteins, essential for metabolism, dynamics, apoptosis, protein import and signaling, are regulated by Ub ligases, including MULAN, membrane‐associated ring finger 5 (MARCH5)/mitochondrial ubiquitin ligase (MITOL) and mitochondria‐associated F‐Box Protein (Mdm) 30, which control fusion and fission processes via ubiquitination (Escobar‐Henriques et al. 2006; Li et al. 2008; Nakamura et al. 2006; Yonashiro et al. 2006). For instance, MARCH5/MITOL ubiquitinates Drp‐1 to regulate membrane assembly and activity rather than targeting it for degradation (Karbowski et al. 2007). Because the OMM lacks dedicated proteases, turnover of many OMM proteins depends mainly on the cytosolic UPS system, especially during proteotoxic stress (Karbowski and Youle 2011; Livnat‐Levanon and Glickman 2011).

Mitochondria can import aggregation‐prone cytosolic proteins and degrade them via matrix proteases (Bertgen et al. 2024; Li et al. 2019; Sutandy et al. 2023). Failure of this system disrupts proteostasis and activates cytosolic stress responses (Mårtensson et al. 2019; Wang and Chen 2015; Weidberg and Amon 2018; Wrobel et al. 2015). As an adaptive mechanism, excess non‐imported mitochondrial precursor proteins can be transiently sequestered into cytosolic structures called MitoStores, thereby limiting their aggregation (Krämer et al. 2023). However, when mitochondrial import stress exceeds the buffering capacity of these protective pathways, precursor proteins accumulate in the cytosol, thus promoting protein aggregation and amplifying proteotoxic stress (Liu, Ma, et al. 2019; Liu, Wang, et al. 2019; Nowicka et al. 2021).

Mitofusins are major targets of proteostatic regulation. In yeast, the mitofusin orthologue Fzo1 is degraded by the 26S proteasome (Escobar‐Henriques et al. 2006). Similarly, Mfn1 and Mfn2 are substrates of the UPS (Anton et al. 2011). After Parkin‐mediated ubiquitination, both are extracted from the OMM via the Vms1‐p97/Cdc48 complex (Tanaka et al. 2010). Under stress conditions, Vms1 translocates to mitochondria and contributes to the OMM protein extraction (Heo et al. 2013). Beyond fusion, Mfn2 also functions as a proteostasis sensor at the mitochondrial surface (Joaquim et al. 2025). Because mitofusins are largely exposed to the cytoplasm, they act as hubs for mitochondria‐cell communication (Escobar‐Henriques et al. 2020; Escobar‐Henriques and Joaquim 2019). Mfn2 is essential for constitutive protein QC, as it interacts with both cytosolic chaperones (HSC70, Hsp90) and the proteasome to prevent protein aggregation, a function not shared by Mfn1 (Joaquim and Escobar‐Henriques 2020). Loss of Mfn2 enhances aggregation, decreases TOM20, TOM70, TOM40 levels and disrupts proteostasis without activating classical cellular stress pathways (Mårtensson et al. 2019; Wang and Chen 2015; Weidberg and Amon 2018; Wrobel et al. 2015). Altered mitochondrial dynamics also affect mitochondrial protein turnover. Opa1‐deficient neurons show increased mitochondrial protein turnover even under oxygen–glucose deprivation and reoxygenation, together with strong upregulation of Oma1, indicating an Oma1‐dependent stress response in addition to fusion defect (Alavi 2021; Fogo et al. 2025). Instead, Drp‐1‐deficient neurons fail to show normal turnover peak during reoxygenation and progressively accumulate damaged proteins (Fogo et al. 2025). Recent evidence shows that, in aged yeast, fission is also activated to segregate specific mitochondrial membrane proteins into mitochondria‐derived compartments for elimination via autophagy (Hughes et al. 2016). Similar structures, known as mitochondria‐derived vesicles (MDVs) have been identified in mammalian cells (Neuspiel et al. 2008; Soubannier et al. 2012), where they participate in antigen presentation and mitophagy‐associated recycling pathways (Ferrucci et al. 2024; Matheoud et al. 2016) (Figure 2D). Recently, an additional form of MQC, termed megamitochondria engulfing lysosomes (MMEL), has been described under hypoxic conditions (Hao et al. 2023). Unlike mitophagy, which eliminates entire dysfunctional mitochondria through the autophagosome–lysosome pathway, and MDVs, which preserve the organelle by exporting, under mild stress, selected mitochondrial cargo to lysosomes for degradation, MMEL promotes localized mitochondrial remodeling by importing lysosomal degradative machinery into megamitochondria while maintaining overall organelle integrity. MMEL have been currently documented primarily under hypoxic stress conditions and, although requiring validation, further expands the repertoire of mechanisms through which cells preserve mitochondrial proteostasis and integrity. Overall, MQC relies on a coordinated hierarchy of mechanisms, including mitochondrial dynamics, MDVs, MMEL, mitophagy, and cytosolic proteostasis pathways such as the UPS, molecular chaperones, ribosome‐associated quality control, and autophagy–lysosomal degradation, to preserve mitochondrial integrity while minimizing unnecessary organelle turnover (Table 1).

### 3.3 The Mitochondrial Unfolded Protein Response: A Temporal Landscape for Mitochondrial Repair

#### 3.3.1 Sensing of Mitochondrial Proteotoxic Stress

The UPRmt is a retrograde proteostatic stress signal from mitochondria to the nucleus, triggering the expression of mitochondrial chaperones, including HspD1, HspE1, and HspA9, and proteases such as LONP1 to restore homeostasis (Münch 2018; Zhao et al. 2002) (Figure 2G). Activation of the UPRmt results from the integration of two major cytosolic stress signals: increased mitochondrial ROS (mtROS) and the cytoplasmic accumulation of inefficiently imported mitochondrial precursor proteins (c‐mtProt) (Sutandy et al. 2023). mtROS oxidize Hsp40 facilitating the recruitment of Hsp70 to precursor proteins and the release of HSF1, which translocates to the nucleus and activates UPRmt genes. Overall, these signaling pathways describe a sophisticated cytosolic surveillance system capable of integrating multiple mitochondrial stress signals, highlighting the close functional connection between mitochondrial and cytosolic proteostasis. In this context, c‐mtProt accumulation remodels cytoplasmic proteostasis (Weidberg and Amon 2018), as shown in *Caenorhabditis elegans* , where disruption of mitochondrial proteostasis induces a coordinated cytosolic response also mediated by lipid biosynthesis (Kim et al. 2016). In nematodes, the central regulator of UPRmt is the stress activated transcription factor (ATFS)‐1 which contains both an N‐terminal mitochondrial targeting sequence and a C‐terminal nuclear localization sequence. Under physiological conditions, ATFS‐1 is imported into the mitochondrial matrix through the TOM and TIM complexes. Once imported, the mitochondrial targeting peptide is cleaved and the protein is degraded by the matrix protease LONP (Nargund et al. 2015), a process requiring ATP; therefore, proper oxidative phosphorylation (OXPHOS) is essential to prevent ATFS‐1 accumulation. During mitochondrial dysfunction, protein import efficiency declines and a fraction of ATFS‐1 remains in the cytosol, subsequently translocating to the nucleus where it activates more than 600 genes involved in proteostasis, mitochondrial biogenesis, and restoration of mitochondrial network function (Nargund et al. 2015). This mechanism allows cells to indirectly monitor mitochondrial functional status. Notably, the mitochondrial targeting sequence of ATFS‐1 is relatively weak compared with those of OXPHOS proteins or mitochondrial chaperones, likely enabling early UPRmt activation before damage becomes irreversible (Rolland et al. 2019; Shpilka et al. 2021). Once mitochondrial import efficiency is restored, ATFS‐1 is again translocated into mitochondria and degraded, thereby contributing to termination of the stress response. In mammals, the functional homolog of ATFS‐1 is ATF5 (Fiorese et al. 2016), which exerts anti‐apoptotic functions through B‐cell lymphoma 2 protein (BCL‐2) expression, similar to HSF1 (Fiorese et al. 2016; Persengiev et al. 2002). ATF4 has also been associated with mitochondrial stress responses and may transcriptionally regulate ATF5 (Melber and Haynes 2018; Quirós et al. 2017).

Mitochondrial protein import is an indicator of mitochondrial proteotoxic stress (Melber and Haynes 2018) and dynamically changes over time: it initially increases during stress to recruit chaperones and protective factors (Poveda‐Huertes et al. 2021; Xin et al. 2022), but progressively declines as damage accumulates (Michaelis et al. 2022). Treatment with the mitochondrial‐targeted Hsp90 inhibitor Gamitrinib‐TPP induced ATF4, C/EBP homologous protein (CHOP) and, subsequently, ATF5 expression, consistent with activation of the UPRmt (Fiesel et al. 2017). This response was accompanied by the accumulation of insoluble proteins within mitochondria, indicating mitochondrial proteotoxic stress (Fiesel et al. 2017). However, ATF4 does not directly activate the UPRmt, but rather contributes to the broader ISR cytoprotective program (Quirós et al. 2017). In line with this, recent proteomic studies have shown that the UPRmt extends beyond mitochondrial chaperones induction and involves broad metabolic and proteomic remodeling to preserve cellular homeostasis during stress (Uoselis et al. 2023). In human cells, an additional mitochondrial stress‐response pathway based on kinase activation has also been identified and likely activated by the mitochondrial anti‐viral signaling protein. The protein kinase RNA‐activated promotes phosphorylation of the transcription factor c‐Jun N‐terminal kinase (JNK2) (Jacobs and Coyne 2013; Rath et al. 2012). JNK2 subsequently activates c‐Jun, a component of the activator transcription factor 1, thereby inducing a nuclear transcriptional response associated with mitochondrial stress (Rath et al. 2012).

#### 3.3.2 Epigenetic and Metabolic Regulation of the UPRmt

UPRmt is also tightly linked to chromatin regulation (Tian et al. 2016). During mitochondrial stress, the cytosolic protein lineage abnormal protein 65 (LIN‐65) translocates to the nucleus in a process dependent on the histone methyltransferase MET‐2, promoting H3K9 histone methylation and global chromatin compaction with transcriptional silencing. Defective proventriculus homolog 1 (DVE‐1)‐regulated genes remain accessible, and become activated (Tian et al. 2016); DVE‐1 activity is further modulated by Ub‐like protein 5 activation (Benedetti et al. 2006) and SUMOylation repression (Gao et al. 2019), which is reversed by Ub‐like protease 4 together with modifications on DVE‐1 and ATFS‐1 (Gao et al. 2019). The mitochondrial matrix ATP‐dependent Clp protease proteolytic subunit 1, also involved in MQC, is also required for LIN‐65 nuclear translocation, reinforcing the close integration between mitochondrial functional status and chromatin organization (Haynes et al. 2007; Mutlu et al. 2018; Tian et al. 2016). UPRmt activation additionally requires the activity of specific demethylases such as homeodomain finger protein 8 and Jumonji C domain‐containing 3 in mammals (Merkwirth et al. 2016), while CREB‐binding protein/E1A binding protein p300 coactivates ATFS‐1‐dependent transcription (Li et al. 2021).

Mitochondrial metabolism further contributes to epigenetics and UPRmt regulation via acetyl‐CoA, oxidized nicotinamide adenine dinucleotide (NAD^+^), and S‐adenosylmethionine, essential substrates for histone acetylation and methylation processes (Mentch et al. 2015; Ryall et al. 2015; Wang et al. 2017). In particular, acetyl‐CoA derived from the Krebs cycle provides the acetyl groups required for histone acetylation (Jo et al. 2020). During mitochondrial stress, reduced Krebs cycle activity lowers intracellular acetyl‐CoA levels, promoting activation of the nucleosome remodeling deacetylase complex and DVE‐1, thereby inducing UPRmt (Zhu et al. 2020).

#### 3.3.3 Systemic Coordination of Mitochondrial Stress Responses

In mammals, stress‐response regulation also involves processing of mitochondrial RNAs. Mitochondrial polycistronic transcripts undergo complex maturation mediated by RNases and RNA‐modifying proteins (Rorbach and Minczuk 2012). The peculiar arrangement of tRNA genes among coding regions requires precise transcript processing by the endonuclease zinc phosphodiesterase 2, responsible for maturation of tRNA 3′ ends, and by the mitochondrial RNase P complex composed of TRNA Methyltransferase 10C, Mitochondrial RNase P Subunit (MRPP)1, MRPP2, and MRPP3, which processes 5′ ends (Rossmanith 2012). Of these, MRPP3 has been identified as a UPRmt target (Münch and Harper 2016). During mitochondrial stress, LONP increases MRPP3 turnover, reducing mitochondrial translation (Münch and Harper 2016).

Beyond intracellular mechanisms, extracellular communication mediated by secreted factors known as mitokines also significantly contributes to mitochondrial homeostasis (Berendzen et al. 2016; Shao et al. 2016; Zhang et al. 2018). These molecules are released by stressed cells and induce non‐cell‐autonomous activation of the UPRmt. In *C. elegans* , neuronal signals induced by mitochondrial stress activate the UPRmt in distant tissues, including intestinal cells. This systemic response also promotes Wnt‐dependent expansion of germline mtDNA copy number, allowing maternal inheritance of elevated mtDNA levels that sustain UPRmt activation and increase stress resistance and longevity in subsequent generations (Zhang et al. 2021).

Overall, the UPRmt represents a key adaptive response that couples mitochondrial proteotoxic stress to mitonuclear signaling programs aimed at restoring mitochondrial proteostasis and preserving cellular homeostasis. Beyond inducing mitochondrial chaperones and proteases, UPRmt activation drives broader transcriptional, proteomic, and metabolic remodeling that adapts mitochondrial function and cellular metabolism to the prevailing stress conditions (Quirós et al. 2017; Münch and Harper 2016). The dependence of UPRmt activation on mitochondrial protein‐import efficiency further enables mitochondrial functional status to be translated into nuclear responses, while mitokine signaling can extend this adaptive response beyond the stressed cell (Nargund et al. 2012; Fiorese et al. 2016). The UPRmt therefore acts not merely as a protein‐folding response, but as a broader mechanism of mitochondrial stress adaptation whose outcome depends on the nature, magnitude, and duration of the stressor. While transient activation can promote recovery and preserve mitochondrial function, persistent or unresolved stress may exceed this adaptive capacity, contributing to the progressive loss of mitochondrial and cellular homeostasis associated with aging and disease.

### 3.4 ER–Mitochondria Contacts: From Proteostasis to Mitochondrial Clearance

#### 3.4.1 Mitochondria‐Associated Membranes as Hubs for Ca^2+^ Signaling and Proteostasis

The physical interface between ER and mitochondria, known as MAMs, is the major communication hub between these two organelles. MAMs are highly dynamic and reversible structures that mediate Ca^2+^ and lipids transfer between ER and mitochondria (van Vliet et al. 2014; Vance 2014). MAMs are particularly enriched in cholesterol, glycosphingolipids, and cardiolipin, and participate in the regulation of lipid metabolism, lipid droplet formation, and cellular energy homeostasis, mitochondrial dynamics, autophagy, apoptosis, and the ER stress response (van Vliet et al. 2014). Among these, the complex formed by the ER‐localized inositol‐1,4,5‐trisphosphate receptor (IP3R), VDAC, and the chaperone glucose‐regulated protein (Grp) 75 supports Ca^2+^ transfer into mitochondria (Szabadkai et al. 2006). DJ‐1 also contributes to this pathway by regulating Ca^2+^ flux toward the IMM through MCU (Basso et al. 2020; Liu, Ma, et al. 2019). Grp75 depletion destabilizes the IP3R–VDAC interaction and reduces ER–mitochondria contacts (Honrath et al. 2017). Another major MAMs regulator is the sigma‐1 receptor, a chaperone that stabilizes IP3R, favors ATP synthesis (Hayashi and Su 2007) and contributes to stress response through IRE1α, and nuclear factor erythroid 2–related factor 2 (NRF2) pathway (Mori et al. 2013; Wang et al. 2015) (Figure 2F). Mitochondrial GTPases Miro1/2 and mitofusins, particularly Mfn2, also participate in ER–mitochondria tethering (Modi et al. 2019). Therefore, alterations in these proteins impair inter‐organelle communication and Ca^2+^ homeostasis.

The ER plays a central role in cellular proteostasis by mediating folding, maturation and QC of proteins entering the secretory pathway. During ER stress, MAMs expand (Schuck et al. 2009) and mitochondria redistribute near to the ER, promoting transfer of Ca^2+^, lipids, and redox signals. Ca^2+^ homeostasis is central to this process because many chaperones involved in protein folding are Ca^2+^ dependent, including calreticulin, Grp94, BiP, and protein disulfide isomerase (Michalak et al. 2009). The calnexin/calreticulin cycle and protein disulfide isomerases also contribute to glycoprotein quality control and Ca^2+^ homeostasis through interaction with sarco/endoplasmic reticulum calcium ATPase and other ion channels (Helenius and Aebi 2004; John et al. 1998). Redox proteins and Ca^2+^ transport systems are enriched at MAMs (Chernorudskiy and Zito 2017; Hamilton et al. 2022; Higo et al. 2005; Li et al. 2009). ER Ca^2+^ channels, including IP3R and ryanodine receptors, cooperate with outer mitochondrial membrane proteins to facilitate mitochondrial Ca^2+^ uptake. Enhanced Ca^2+^ transfer supports oxidative phosphorylation and ATP production (Katona et al. 2022). Within these microdomains, PERK and endoplasmic reticulum oxidoreductin (ERO) 1α regulate mitochondrial dynamics and energy metabolism (Bassot et al. 2023), while ATF4 controls genes involved in amino acid metabolism, redox homeostasis, and mitochondrial function (Harding et al. 2003). However, persistent ER stress may lead to excessive mitochondrial Ca^2+^ accumulation, opening of the mitochondrial permeability transition pore and cell death (Pinton et al. 2008; Puthalakath et al. 2007). In parallel, ATF4 induces CHOP, which upregulates ERO1α expression and ROS production, promoting mitochondrial dysfunction and apoptosis (Puthalakath et al. 2007). Therefore, the PERK–ATF4–CHOP–ERO1α axis amplifies these effects by inducing ROS generation, mitochondrial dysfunction, and expression of pro‐apoptotic BCL‐2 family proteins (Puthalakath et al. 2007).

#### 3.4.2 Mitochondria‐Associated Membranes Coordinate Autophagy and Mitochondrial Quality Control

In recent years, MAMs have also emerged as central platforms of autophagy. Although the origin of autophagosomal membranes remained controversial for many years, recent evidence indicates that, in mammalian cells, autophagosomes predominantly form at ER–mitochondria contact sites (Hamasaki et al. 2013), which expand during cellular stress (Csordás et al. 2006; Tagaya and Arasaki 2017). Autophagy is mainly activated during nutrient deprivation through unc‐51 like autophagy activating kinase 1 (ULK1) and involves several MAM‐localized proteins, including autophagy related (ATG)14, ATG2, WD repeat domain phosphoinositide‐interacting protein 1 (WIPI1), Beclin‐1, and AMBRA1 (Garofalo et al. 2016; Grimmel et al. 2015; Tang et al. 2019). During starvation, ATG14, a specific component of the phosphoinositide 3‐kinase complex involved in the early stages of autophagosome biogenesis (Matsunaga et al. 2009, 2010), accumulates at MAMs together with double FYVE domain containing protein 1 and ATG5 (Axe et al. 2008; Hayashi‐Nishino et al. 2009). ATG5 remains associated with MAMs throughout autophagosome membrane expansion, indicating that these regions function as dynamic platforms for autophagosome formation (Hamasaki et al. 2013). Disruption of ER–mitochondria contacts through depletion of phosphofurin acidic cluster sorting protein 2 or Mfn2 impairs recruitment of these proteins and reduces autophagosome formation (de Brito and Scorrano 2008; Simmen et al. 2005). The soluble N‐ethylmaleimide‐sensitive factor attachment protein receptor (SNARE) protein syntaxin‐17 (STX17) also accumulates at MAMs during starvation and directly interacts with ATG14 in autophagosome maturation (Hamasaki et al. 2013). Loss of STX17 prevents proper autophagosome maturation and leads to accumulation of immature isolation membranes, highlighting the dependence of autophagic trafficking on functional MAM organization (Hamasaki et al. 2013). Nevertheless, the mechanisms by which membranes and lipids are transferred from the ER and mitochondria to the growing phagophore remain undeciphered. Another MAM‐associated protein involved in autophagy is AMBRA1, which is inactive under basal conditions but becomes phosphorylated by ULK1 during starvation, translocates to the ER, and cooperates with the Beclin‐1 complex in autophagosome formation (Antonioli et al. 2014; Di Bartolomeo et al. 2010; Di Rienzo et al. 2019; Maria Fimia et al. 2007; Nazio et al. 2013). At MAMs, AMBRA1, together with Beclin‐1, interacts with the ganglioside GD3 and WIPI1 to remodel membranes during autophagosome biogenesis (Garofalo et al. 2016). MAMs also contain lipid raft‐like microdomains enriched in cholesterol and sphingolipids (Lingwood and Simons 2010; Poston et al. 2011; Rowland and Voeltz 2012) that host proteins including thioredoxin‐related transmembrane protein 1, sigma non‐opioid intracellular receptor 1, presenilin‐2, and reticulon 1 (Hayashi and Fujimoto 2010; Lynes et al. 2012; Reali et al. 2015). ER lipid raft associated (ERLIN)1 and ERLIN2, associated with these domains (Browman et al. 2006; Pednekar et al. 2011), participate in IP3R degradation, cholesterol metabolism, and control of mitochondrial function (Pearce et al. 2009; Wang et al. 2012). During starvation, ERLIN1 interaction with AMBRA1 promotes autophagy activation (Manganelli et al. 2021). This interaction depends both on the structural integrity of ER–mitochondria contacts and on the lipid composition of MAMs, which is also influenced by Mfn2 and the ganglioside GD3 (Cosson et al. 2012; de Brito and Scorrano 2008; Garofalo et al. 2016; McLelland and Fon 2018). Collectively, MAMs constitute dynamic platforms integrating Ca^2+^ homeostasis, metabolism, proteostasis, stress responses, and autophagy, and coordinating cellular adaptation and cell fate (Table 1).

Altogether, the processes discussed in this section act at multiple levels to preserve mitochondrial and cellular homeostasis. MQC mechanisms control mitochondrial renewal and the selective removal of damaged organelles or components; the UPRmt provides an adaptive response to mitochondrial proteotoxic stress by reshaping protein folding, degradation, metabolism, and gene expression; and ER–mitochondria contacts coordinate signaling and metabolic exchanges while also supporting autophagic and mitochondrial clearance. The relevance of these mechanisms lies also in their ability to limit the persistence and propagation of mitochondrial damage. In neurons, where mitochondrial function and proteostasis must be preserved over long periods, failure of these protective responses can favor the accumulation of dysfunctional mitochondria and misfolded proteins, increase oxidative and metabolic stress, and compromise organelle turnover. These alterations create a permissive environment for the reciprocal amplification of mitochondrial dysfunction and failure of proteostasis that characterizes neurodegeneration, which is addressed in the next section.

## 4 Proteostasis and Mitochondrial Quality Decline: Synergistic Pathological Features in Neurodegeneration

### 4.1 Mitochondrial Quality Control and Proteostasis Dysfunction in Neurodegeneration

**FIGURE 3:**
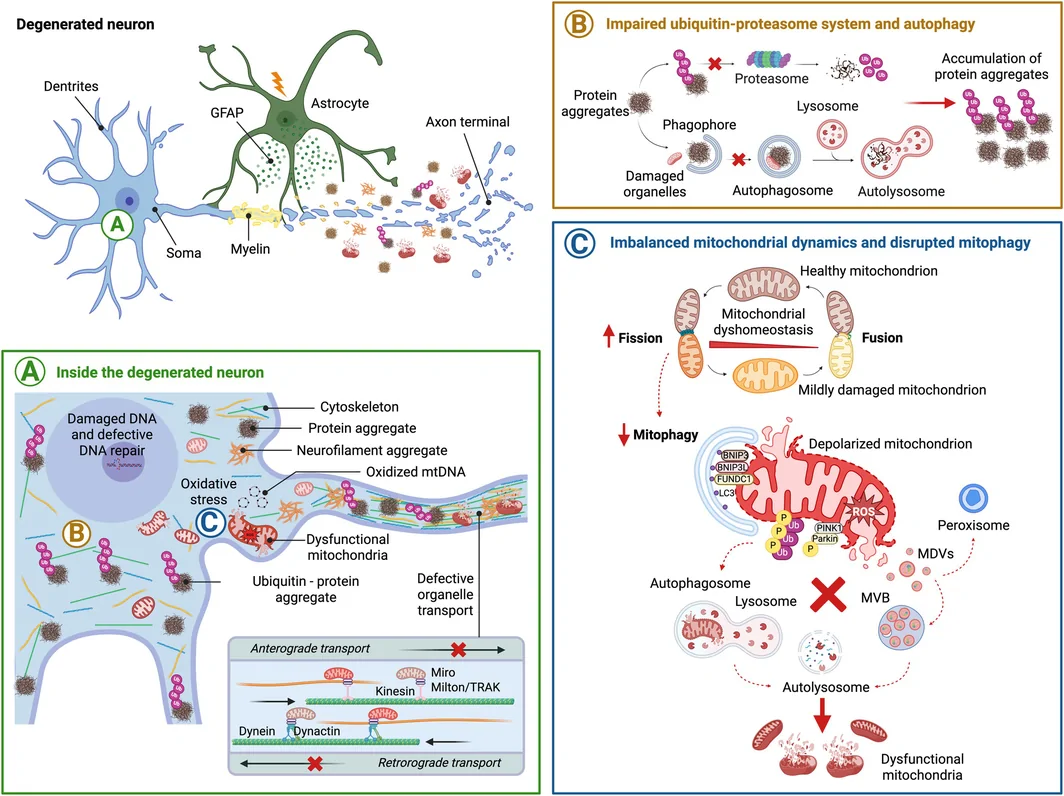
Schematic representation of a degenerated neuron and the dysfunctional processes associated with neurodegeneration. (A) A degenerating neuron is morphologically characterized by an atrophic soma, reduced dendritic spine density, axonal fragmentation, myelin damage, and extracellular release of aberrant protein aggregates and dysfunctional mitochondria. (B) Aberrant protein aggregates accumulate due to impaired ubiquitin‐proteasome and autophagic systems. (C) The accrual of dysfunctional mitochondria is the result of an inefficient mitochondrial quality control system characterized by imbalanced mitochondrial dynamics producing a high amount of fissioned organelles that are inadequately removed by impaired mitophagy. Abbreviations: BNIP3, BCL2/adenovirus E1B 19‐kDa protein‐interacting protein 3; BNIP3L, BNIP3 like; FUNDC1, FUN14 domain‐containing 1; GFAP, glial fibrillary acidic protein; LC3, microtubule‐associated protein 1A/1B‐light chain 3; MBV, multivesicular bodies; MDV, mitochondria‐derived vesicle; Miro, mitochondrial Rho GTPase; mtDNA, mitochondrial DNA; MVB, multivesicular bodies; P, phosphate residue; PINK1, PTEN‐induced kinase‐1; ROS, reactive oxygen species; TRAK, trafficking kinesin protein; Ub, ubiquitin; UPS, ubiquitin‐proteasome system. Created with BioRender.com.

Aberrant proteostasis and altered energy homeostasis are recognized as two interconnected hallmarks of neurodegeneration, reflecting the intimate relationship between protein homeostasis and mitochondrial function in neuronal health and disease (Wilson et al. 2023). Accordingly, proteostasis and MQC cooperate to preserve neuronal homeostasis, and their progressive dysfunction establishes a feed‐forward cycle that amplifies mitochondrial impairment, protein aggregation, and ultimately neurodegeneration (Figure 3A–C).

Mitophagy decline, triggered by Parkin/PINK1‐related mutations, is tightly linked to the accrual of ROS‐producing mitochondria in neurodegeneration (Xiao et al. 2022) (Figure 3C). This can impinge on neuronal activity by inflicting oxidative damage and overwhelming the PN (Pytel and Fromm Longo 2025). Failure of the PN largely contributes to the accumulation of misfolded proteins and loss of synaptic plasticity, thus facilitating the initiation and progression of proteotoxic‐associated disorders (Figure 3B). Therefore, protein aggregation can directly affect mitochondrial function.

For instance, α‐synuclein can interfere with mitochondrial function through multiple mechanisms. The A53T mutant can localize at the IMM and directly impair mitochondrial electron transport chain activity (Chinta et al. 2010; Devi et al. 2008) as well as impinge on MQC via mitochondrial dynamics (Thorne and Tumbarello 2022). In Parkinson's disease, pathogenic α‐synuclein can also interfere with mitochondrial protein import by binding TOM20 and disrupting its interaction with TOM22, thereby impairing the entry of nuclear‐encoded proteins into mitochondria and compromising mitochondrial function (Di Maio et al. 2016). Consistent with a broader impairment of MQC, post‐mortem analyses of dopaminergic neurons from patients with Parkinson's disease revealed coordinated alterations in pathways involved in mitophagy and mitochondrial proteostasis, suggesting a reduced capacity to maintain and recycle the mitochondrial pool (Chen et al. 2023). As a result, this leads to a bioenergetic failure that is detrimental to neuronal activity.

Alterations in mitochondrial proteolysis provide an additional link between mitochondrial proteostasis and neurodegeneration. Intraneuronal Aβ42 interacts with the mitochondrial matrix protease LONP1 and impairs its proteolytic activity, contributing to mitochondrial proteostasis deficits and mitochondrial dysfunction in the brains of mice and humans with Alzheimer's disease (Wang et al. 2023). Restoration of LONP1 expression ameliorated mitochondrial abnormalities and cognitive deficits in CRND8 APP transgenic mice (Wang et al. 2023). Mitochondrial proteases can also influence organelle remodeling and neuronal survival. Stress‐induced OMA1 activation promotes OPA1 processing, limiting mitochondrial fusion and promoting neuronal death, whereas genetic ablation of OMA1 stabilized fusion‐active long OPA1 forms, delayed neuronal loss, and prolonged survival in a mouse model of neurodegeneration caused by neuronal prohibitin deficiency (Korwitz et al. 2016).

Alterations in inter‐organelle communication provide an additional mechanism linking mitochondrial dysfunction to neurodegeneration. Mutated presenilin‐1, a γ‐secretase catalytic subunit implicated in familial Alzheimer's disease, localizes at mitochondria and affects mitochondrial function and homeostasis (Han et al. 2021). This occurs via MAMs and the ensuing oxidative stress contributes to premature disease onset in mutation carriers (Han et al. 2021). Mitochondrial alterations often precede tissue pathology, indicating that the imbalance of mitochondrial quality influences synaptic development and plasticity before neuronal death (Kural et al. 2026).

### 4.2 Therapeutic Targeting of Mitochondrial Quality Control and Proteostasis

Considering the central role of mitochondrial failure to cellular quality and homeostasis, mitochondria transplantation has been proposed as a rescue strategy to alleviate mitochondrial dysfunction in multiple settings, including models of Parkinson's disease and conditions characterized by mitochondrial disorders (Du et al. 2026). Although effective strategies for delivering exogenous mitochondria to somatic cells and tissues remain under development, the transfer of mitochondria within vesicles derived from the plasma membrane of erythrocytes seems to be a valuable strategy (Du et al. 2026). This has been shown to successfully restore mitochondrial function in patient‐derived cells carrying mtDNA deletions, mutations, and/or depletion, rescuing associated bioenergetic and metabolic defects (Du et al. 2026). In a murine model of Parkinson's disease, mitochondrial transfer reduced neuronal loss, restored mitochondrial activity in affected brain regions, and improved motor performance (Du et al. 2026).

Additional promising therapeutic options are those based on the treatment with NAD^+^ precursors to boost both the UPRmt and mitophagy (Zhou et al. 2025). NAD augmentation attenuates motor deficits and delays neuropathological progression in 1‐methyl‐4‐phenyl‐1,2,3,6‐tetrahydropyridine‐induced models of Parkinson's disease through mechanisms dependent on the ATF4‐mediated UPRmt. Integrated transcriptomic and metabolomic analyses of the striatum further showed that nicotinamide mononucleotide induces large molecular changes in pathways involved in mitochondrial homeostasis and QC. Collectively, these findings indicate that impaired MQC contributes to the accelerated pathology observed in Parkinson's disease models, while increasing cellular NAD^+^ levels alleviates mitochondrial proteotoxic stress and mitigates disease‐associated phenotypes (Zhou et al. 2025). The translational potential of NAD^+^ replenishment has also been explored in patients with Parkinson's disease. In the NADPARK Phase I trial, nicotinamide riboside supplementation was well tolerated, increased cerebral NAD levels, and induced molecular changes involving mitochondrial, lysosomal, and proteasomal processes, providing initial evidence that NAD^+^ increase can engage pathways relevant to cellular QC in humans (Brakedal et al. 2022). Although a recent study reported that NAD^+^ levels do not vary across age groups and lifestyles, the possibility that pathological NAD^+^ deficiencies may arise under metabolic and/or degenerative conditions cannot be ruled out and may provide a rationale for NAD^+^‐boosting interventions (Trętowicz et al. 2026).

Other therapeutic approaches target mitochondrial turnover and remodeling more directly. Urolithin A, a mitophagy‐promoting metabolite, improved cognitive performance and reduced Aβ and tau pathology in multiple mouse models of Alzheimer's disease, while restoring mitophagy and lysosomal function (Hou et al. 2024). Modulation of mitochondrial dynamics represents another potential strategy. Pharmacological inhibition of excessive DRP1‐dependent fission ameliorated synaptic dysfunction, Aβ deposition, and cognitive impairment in a double‐transgenic mice model of Alzheimer's disease (Baek et al. 2017), supporting the possibility that restoring the balance of mitochondrial dynamics may preserve neuronal function. Therapeutic modulation of proteostasis may also extend beyond mitochondria. Spermidine, which promotes autophagy, has shown neuroprotective effects in experimental settings, although translation to humans remains uncertain: a 12‐month randomized trial in older adults with subjective cognitive decline did not improve the primary memory outcome (Schwarz et al. 2022). Finally, modulation of MAM integrity has been proposed as a potential strategy to restore the spatial coordination of organelle homeostasis in neurodegeneration, although its therapeutic potential remains to be established (Galizzi 2026).

Together, these observations indicate that targeting MQC and proteostasis can act at complementary levels, from enhancing protein and organelle clearance to restoring mitochondrial remodeling. At the same time, the discrepancy between encouraging preclinical findings and the still limited clinical evidence underlies the challenges associated with translating modulation of these interconnected processes into effective therapies for neurodegenerative diseases.

## 5 Conclusion

MQC processes involve multiple mechanisms to repair, remodel, renew, or eliminate damaged mitochondrial components and/or entire organelles, while stress responses and inter‐organelle communication extend this surveillance beyond mitochondria. The progressive impairment of these processes can favor the accumulation of dysfunctional mitochondria and misfolded proteins, oxidative and metabolic stress, and defective organelle turnover, thereby contributing to neuronal vulnerability and neurodegeneration. The complementary nature of MQC pathways has important implications for therapeutic development. Effective interventions may need to be developed to preserve the balance between mitochondrial repair, replacement, and removal according to the extent and nature of mitochondrial damage, rather than targeting individual pathways. Despite substantial progress, several unanswered questions remain regarding what determines the capacity of the mitochondrial network to recover and when this capacity becomes irreversibly compromised. A key issue is whether a critical threshold of functional mitochondrial integrity exists below which endogenous repair and biogenesis can no longer restore mitochondrial homeostasis. Equally important is understanding how proteostatic responses, organelle repair, mitophagy, and biogenesis are coordinated and prioritized as mitochondrial damage increases, and when this coordination becomes insufficient to restore homeostasis. Finally, how cells transition from repairing individual proteins or mitochondrial components to eliminating entire organelles, and how these decisions are altered during aging and neurodegeneration remain to be established.

## Author Contributions

**Rosa Di Lorenzo:** conceptualization, writing – original draft. **Rola S. Zeidan:** writing – review and editing. **Vito Pesce:** writing – review and editing. **Emanuele Marzetti:** supervision, writing – review and editing. **Anna Picca:** conceptualization, supervision, writing – review and editing. **Riccardo Calvani:** writing – review and editing.

## Funding

This work was supported by the Università Cattolica del Sacro Cuore (D1.2024 and D1.2025), the nonprofit research foundation Centro Studi Achille e Linda Lorenzon (N/A), and the Italian Ministry of Health (Ricerca Corrente 2026).

## Conflicts of Interest

The authors declare no conflicts of interest.

## Data Availability

Data sharing not applicable to this article as no datasets were generated or analysed during the current study.
